# Non-canonical *BRAF* variants and rearrangements in hairy cell leukemia

**DOI:** 10.32604/or.2024.051218

**Published:** 2024-08-23

**Authors:** STEPHEN E. LANGABEER

**Affiliations:** Cancer Molecular Diagnostics, St. James’s Hospital, Dublin, D08 W9RT, Ireland

**Keywords:** Hairy cell leukemia, *BRAF*, Molecular diagnostics, Targeted therapy

## Abstract

Hairy cell leukemia (HCL) is an uncommon mature B-cell malignancy characterized by a typical morphology, immunophenotype, and clinical profile. The vast majority of HCL patients harbor the canonical *BRAF* V600E mutation which has become a rationalized target of the subsequently deregulated RAS-RAF-MEK-MAPK signaling pathway in HCL patients who have relapsed or who are refractory to front-line therapy. However, several HCL patients with a classical phenotype display non-canonical *BRAF* mutations or rearrangements. These include sequence variants within alternative exons and an oncogenic fusion with the *IGH* gene. Care must be taken in the molecular diagnostic work-up of patients with typical HCL but without the *BRAF* V600E to include investigation of these uncommon mechanisms. Identification, functional characterization, and reporting of further such patients is likely to provide insights into the pathogenesis of HCL and enable rational selection of targeted inhibitors in such patients if required.

## Hairy Cell Leukemia

Classical Hairy Cell leukemia (HCL) is an uncommon B-cell malignancy morphologically characterized by the typical presence of medium-sized, mature B-lymphocytes with cytoplasmic, villous projections in the bone marrow and spleen. HCL cells usually express CD11c, CD20, CD25, and CD103 allowing a relatively rapid diagnosis of suspected cases by immunophenotyping. HCL is more prevalent in men than women with common clinical signs including recurrent infection, splenomegaly, and pancytopenia with moncytopenia prevalent [1]. The standard first-line therapy for HCL is a purine nucleoside analog (cladribine or pentostatin) with or without the chimeric monoclonal antibody rituximab that targets CD20. However, a significant number of patients, almost half, will relapse or will become refractory and require further, alternative lines of treatment [2].

## *BRAF* V600E Mutation

A milestone in the pathobiology of HCL was the discovery of the *BRAF* V600E mutation (c.1799T>A; NM_004333.4) in nearly all cases of the classical form of HCL over a decade ago which was achieved by whole exome sequencing [3]. The RAS-RAF-MEK-MAPK intracellular signaling pathway is one of the most commonly mutated oncogenic pathways in cancer with the *BRAF* V600E mutation previously described at a high frequency in malignant melanoma, papillary thyroid cancer, and colorectal cancer [4]. Transplantation of *BRAF* V600E hematopoietic stem cells (HSC) into mice results in stable engraftment, revealing the functional self-renewable capacity of HCL HSC. Forced expression of the oncogene in murine HSC results in a lethal hematopoietic disorder but restricting expression to mature B cells does not result in disease [5]. Given that HCL cells display a gene expression signature similar to that of post-germinal center B cells, there is an implication that additional genetic alterations are co-operatively required to induce hairy cell development from B cells [6]. Recent murine studies have shown that concurrent mutations in tumor suppressors such as *TP53* and *PTEN* are required for HCL ontogeny [7]. Despite the high frequency of the *BRAF* V600E in HCL [8–10] corroborated by several groups [11–14], there remained some patients with classical HCL in whom the *BRAF* V600E mutation could not be detected [15,16].

## Non-Canonical *BRAF* Mutations and Rearrangements

In those HCL patients in whom no *BRAF* V600E mutation could be detected, alternative molecular mechanisms that activate the RAS-BRAF-MEK-MAPK pathway in capitulating the HCL phenotype are likely involved. Cases were identified by a National Library of Medicine search (https://pubmed.ncbi.nlm.nih.gov/). Sequencing all of *BRAF* exon 15 and alternative exons has demonstrated the presence of other mutations that result in HCL (Table 1) [17–19]. It is noteworthy that the V600E mutation is co-existent in three cases.

**Table 1:** Non-canonical *BRAF* mutations and rearrangements in classical HCL

Patient	Reference	Sex	Age	Non-canonical *BRAF* mutation/rearrangement		Additional *BRAF* V600E
1	Tschernitz et al. [17]	M	68	D449E	exon 11	No
2	Tschernitz et al. [17]	M	64	F468C	exon 11	No
3	Tschernitz et al. [17]	M	59	S602T	exon 15	Yes
4	Thomas et al. [18]	U	U	K601T	exon 15	Yes
5	Maitre et al. [19]	M	57	F595L	exon 15	No
6	Maitre et al. [19]	M	39	W604L	exon 15	Yes
7	Thompson et al. [20]	M	44	t(7;14) (q34;q32)	*IGH* (JH)-*BRAF* (exon 10)	No
8	Matsumoto et al. [21]	M	60	t(7;14) (q34;q32)	*IGH* (JH)-*BRAF* (exon 10)	No

**Figure 1:**
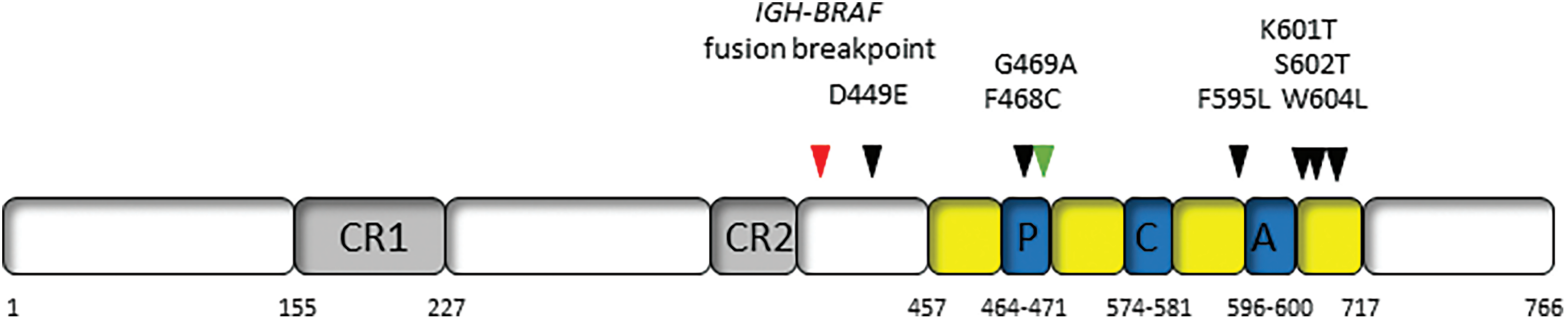
Location of non-canonical *BRAF* mutations and rearrangements in hairy cell leukemia. CR1 and CR2: highly conserved regulatory regions; P: P loop; C: catalytic loop; A: activation loop.

In addition to these mutations, in two *BRAF* V600E-negative HCL patients, an *IGH-BRAF* translocation was detected by fluorescent *in situ* hybridization (FISH) [20,21] (Table 1). The translocation occurs in the *IGM* switch region of the *IGH* locus, which is also interrupted in *IGH-MYC* and *IGH-BCL6* fusions, that fuses to *BRAF* exon 10 [20,21] (Fig. 1). Both patients with this translocation showed molecular evidence of RAS-BRAF-MEK-MAPK pathway activation.

## Molecular Diagnostics

Characterization of the *BRAF* V600E mutation has now become an integral part of the diagnostic workup for suspected HCL [22,23] but recognition of these non-canonical mutations and rearrangements has implications for the molecular diagnostic approach employed. While techniques such as allele-specific quantitative PCR or droplet digital PCR can sensitively identify the V600E and consequently monitor disease burden [24,25] they will not expose *BRAF* variants in other exons. Consideration must therefore be given to include sequencing of at least *BRAF* exon 11 (in addition to exon 15), if not all coding exons of *BRAF* in V600E-negative cases. While this would only be an occasional practice, such sequencing approaches would need to be validated with the use of appropriate internal and external quality control. FISH with an *IGH* probe is also indicated in those cases of HCL that have a classical morphology, clinical features, and immunophenotype. The time and expense of such further investigations would be easily offset by the potential discovery of a non-canonical *BRAF* mutation that would allow appropriately selected treatment with an inhibitor or not.

## *BRAF* Pathway Targeted Therapy

**Figure 2:**
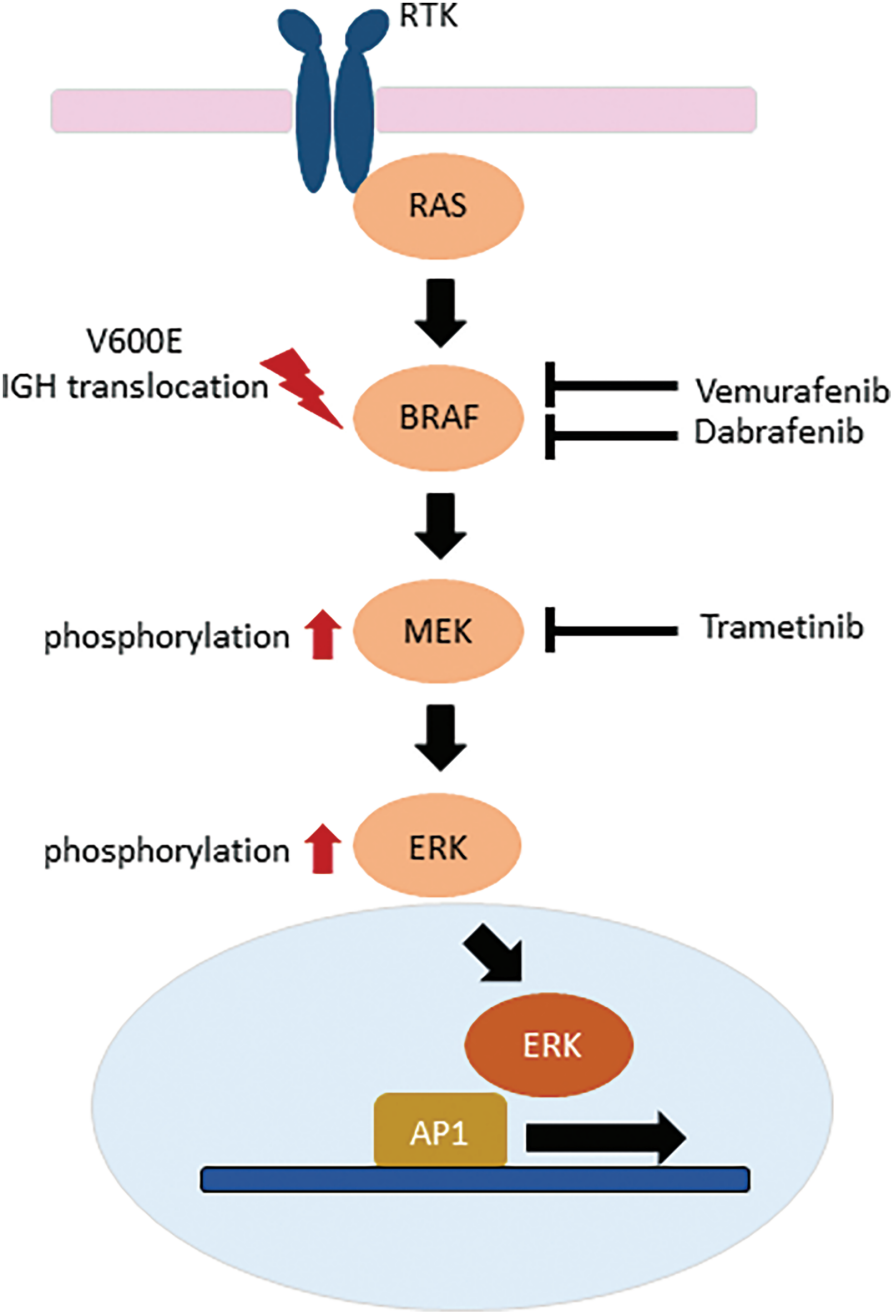
Inhibitors of the RAS-RAF-MEK-ERK intracellular signaling pathway in relapsed or refractory hairy cell leukemia.

Given the high rate of relapse with standard front-line therapy, the molecular defect resulting from the *BRAF* V600E is a highly attractive target of therapy in HCL patients with relapsed or refractory disease. As the *BRAF* inhibitor Vemurafenib was already available for *BRAF* V600E-mutated melanoma [26], proof of principle for clinical application in relapsed/refractory HCL was rapidly proven followed by that of Dabrafenib [27–29]. In HCL cells, *BRAF* inhibitors cause marked MEK/ERK dephosphorylation, silencing of the RAS-RAF-MEK-MAPK pathway, loss of the HCL-specific gene expression signature, and eventually apoptosis [30]. Clinical trials ensued demonstrating the efficacy of a short oral course of Vemurafenib. However, the persistence of phosphorylated ERK leukemia cells at the end of treatment suggested bypass reactivation of MEK and ERK as a resistance mechanism [31,32]. Specific inhibition of MEK activity is also a therapeutic option [33] with combinations of inhibitors of this same pathway currently being explored [34] (Fig. 2).

How therefore do the above-described non-canonical *BRAF* mutations and rearrangement potentially impact targeted therapy? Amongst all cancers, a wide range of *BRAF* mutations have been described and can be divided into three classes based on biochemical and signaling aspects though this classification remains controversial. Class I mutations are within the V600 codon and result in strong kinase activity; Class II mutations are non-V600 variants that have weaker downstream kinase activity; and Class III mutations result in very low kinase activity and cannot directly phosphorylate MEK [35]. Given their location in exon 15, outside the activation loop (Fig. 1) and proximity to codon V600, the K601T, S602T and W604L would be likely categorized as Class II mutations displaying some response to *BRAF* and *MEK* inhibitors, whereas the exon 11 mutations of D449E and F468C might be considered Class III with limited response to inhibitors of this signaling pathway. Caution must be taken as the functional characteristics of each of these mutations have not been demonstrated *ex vivo*. The patients with *IGH-BRAF* fusions were treated with cladribine and rituximab and cladribine only, achieving long-term molecular and clinical responses [20,21]. However, in one of the latter patients, HCL cells harboring the *IGH-BRAF* fusion were resistant to Vemurafenib with the authors suggesting it may be advisable not to administer *BRAF* inhibitors to such patients [21].

While this review has focused on non-canonical abnormalities in patients with classical HCL, such a mutation has been recently described in a patient with the variant form of HCL (CD25-negative). The *BRAF* G469A is within the protein kinase domain which results in increased *BRAF* dimerization, kinase activity, and ERK activation [36].

## Conclusions

Testing for the presence of the *BRAF* V600E mutation is necessary for patients with relapsed or refractory HCL to assign appropriate inhibitor therapy. The above-described non-canonical means of *BRAF* de-regulation must in some form resemble that of the V600E as they all result in an HCL phenotype. Identification, further functional characterization, and reporting of more HCL patients with non-canonical mutations and rearrangements are required to better understand how they disrupt the individual components of the RAS-RAF-MEK-MAPK signaling pathway and provide an opportunity for rationalized selection of inhibitors.

## Data Availability

Not applicable.
